# Evaluation of Elekta Agility multi‐leaf collimator performance using statistical process control tools

**DOI:** 10.1002/acm2.12660

**Published:** 2019-06-14

**Authors:** Sandra M. Meyers, Michael J. Balderson, Daniel Létourneau

**Affiliations:** ^1^ Radiation Medicine Program Princess Margaret Cancer Center Toronto Ontario Canada; ^2^ Department of Radiation Medicine and Applied Sciences, Moores Cancer Center University of California, San Diego La Jolla California; ^3^ Department of Radiation Oncology University of Toronto Toronto Ontario Canada

**Keywords:** control charts, multi‐leaf collimator, quality control, SBRT treatment delivery, statistical process control

## Abstract

**Purpose:**

To evaluate the performance and stability of Elekta Agility multi‐leaf collimator (MLC) leaf positioning using a daily, automated quality control (QC) test based on megavoltage (MV) images in combination with statistical process control tools, and identify special causes of variations in performance.

**Methods:**

Leaf positions were collected daily for 13 Elekta linear accelerators over 11‐37 months using the automated QC test, which analyzes 23 MV images to determine the location of MLC leaves relative to radiation isocenter. Leaf positioning stability was assessed using individual and moving range control charts. Specification levels of ±0.5, ±1, and ±1.5 mm were tested to determine positional accuracy. The durations between out‐of‐control and out‐of‐specification events were determined. Peaks in out‐of‐control leaf positions were identified and correlated to servicing events recorded for the whole duration of data collection.

**Results:**

Mean leaf position error was −0.01 mm (range −1.3–1.6). Data stayed within ±1 mm specification for 457 days on average (range 3–838) and within ±1.5 mm for the entire date range. Measurements stayed within ±0.5 mm for 1 day on average (range 0–17); however, our MLC leaves were not calibrated to this level of accuracy. Leaf position varied little over time, as confirmed by tight individual (mean ±0.19 mm, range 0.09–0.43) and moving range (mean 0.23 mm, range 0.10–0.53) control limits. Due to sporadic out‐of‐control events, the mean in‐control duration was 2.8 days (range 1–28.5). A number of factors were found to contribute to leaf position errors and out‐of‐control behavior, including servicing events, beam spot motion, and image artifacts.

**Conclusions:**

The Elekta Agility MLC model was found to perform with high stability, as evidenced by the tight control limits. The in‐specification durations support the current recommendation of monthly MLC QC tests with a ±1 mm tolerance. Future work is on‐going to determine if performance can be optimized further using high‐frequency QC test results to drive recalibration frequency.

## 1 INTRODUCTION

Accurate delivery of conformal and intensity modulated radiation treatments (IMRT) is highly dependent on multi‐leaf collimator (MLC) leaf positioning accuracy. This is especially important for stereotactic body radiation therapy (SBRT), where high doses of radiation are delivered to targets with small setup margins, which are on the order of mechanical machine specifications.^1^, ^2^, ^3^, ^4^, ^5^

In order to ensure accuracy and precision of MLC leaf positioning, routine MLC quality control (QC) testing is recommended to be performed weekly using visual inspection of matched segments and monthly quantitative testing using a procedure such as a picket fence test, with a tolerance of 1 mm.^1^ However, it is possible that a higher frequency of quantitative testing, in combination with a high accuracy test, could enable MLC units to perform to a tighter tolerance. Currently, accurate MLC testing is time‐consuming, which limits the feasibility of higher frequency testing in a clinical setting. Streamlining quantitative MLC QC and the results analysis using automated tools enables performance assessment and is the first step toward MLC performance optimization.

A previous study introduced an automated QC test for MLC leaf positioning accuracy,^6^ which was performed three to four times per week on two units to assess the performance of the Elekta MLCi and MLCi2 Elekta MLC models. The purpose of this work was to apply this test daily, along with statistical process control tools, to evaluate the long‐term performance and stability of the Elekta Agility MLC model. Control charts were used to characterize normal behavior, in order to detect abnormal or special cause variations. A number of factors were investigated to determine the cause of variation in MLC leaf position, including leaf positioning mechanism, servicing events, and beam steering. The impact of image artifacts on MLC leaf position was also assessed, as the automated QC test in this work uses megavoltage (MV) imaging to measure leaf positions. This study will provide the groundwork needed to implement prospective, automated MLC QC and control‐chart based analysis in order to optimize MLC performance.

## 2 METHODS

Leaf positions were collected daily for 13 Elekta units over 11–37 (average 22) months using the automated QC test, which analyzes 23 MV images to determine the location of MLC leaves relative to the radiation isocenter.^6^ First, the location of the radiation isocenter is detected using 9 MV images of a 4 × 4 cm^2^ field acquired at various collimator angles. Then the relative panel and collimator angular alignment is estimated from the position of leaf pairs extending into a 20 × 20 cm^2^ radiation field. Finally, leaf positions are measured for five different pickets (4 × 24 cm^2^ fields) located at five nominal leaf‐bank positions. Picket 1 consists of Y1 leaves at nominal −60 mm and Y2 leaves at 100 mm. Images for each picket are acquired at collimator 0° and 180° to capture all MLC leaves in the MV imager field‐of‐view. A 6 MV beam is used for all images. In total, extending the imaging panel, running the beam, collecting the images, and performing the analysis takes about 7–8 minutes and requires no user intervention after starting the first beam. More details about the leaf measurement procedure can be found in the work of Létourneau et al.^6^

A pair of individual and moving range control charts^7^ was produced for each leaf to assess long‐term leaf positioning reproducibility and stability of the system. Individual control limits correspond to approximately three times the standard deviation of measured leaf positions on either side of the mean leaf position. Moving range is computed as the absolute value of the difference in a measurement value from the measurement prior. The lower moving range control limit is 0, and the upper limit is 3.268 times the mean moving range, which corresponds to three times the population standard deviation. This means there is a 0.27% chance of observing a measurement outside of these control limits due to normal variations in the process. While the individual control chart highlights the fluctuations in the measured statistic’s mean (i.e., leaf position) over time, the moving range control chart detects changes in the process variance and emphasizes the rate of change of leaf positions as a function of time. Together, these charts are used to demonstrate whether a process variation is in or out of control. Control limits were computed using MATLAB, and were recomputed following MLC recalibration. MLC recalibration was performed using the vendor’s recalibration procedure, which takes about 60 minutes.

Leaf position error was computed as actual position minus nominal, where a negative error indicates the leaf extended further than the nominal position. Specification levels of ±0.5, ±1, and ±1.5 mm were tested to determine the MLC system’s positional accuracy. The mean and range of duration between out‐of‐control and out‐of‐specification events (i.e., measurements falling outside of control and specification limits, respectively) were determined.

Beam spot motion could affect measured leaf positions by shifting the projection of the MLC on the imager by ray lines originating from the beam spot. Although the MLC leaves would not actually be moving in space, the radiation field edges defined by the MLC would be shifted. In order to assess whether beam spot motion was affecting leaf position errors, daily differences (current minus prior leaf position) were computed for each measured leaf position, and averaged over all leaves in each bank. A Pearson’s correlation coefficient was computed between Y1 and Y2 mean daily differences for each picket of each unit. A negative correlation would indicate that both Y1 and Y2 leaves were appearing to shift in the same direction on a day‐to‐day basis, which would occur if the beam itself was moving. For units that had recalibrations, separate correlation coefficients were computed for leaf positions before and after the recalibration.

Control chart limits were used to identify apparent abnormal MLC leaf behavior. To quantitatively identify changes in leaf position control, the number of moving range out‐of‐control leaf positions (summed over all 160 leaves and all pickets for each measurement day) was plotted over time. Moving range out‐of‐control events were selected because they highlight shifts to out‐of‐control behavior, whereas individual out‐of‐control events demonstrate a continuous out‐of‐control state. Large peaks in out‐of‐control leaf positions were identified as having amplitude greater than the mean plus 1 standard deviation in the number of out‐of‐control leaf positions. Servicing events were recorded for each unit and plotted against the identified peaks in out‐of‐control points to assess correlation. Servicing events that fell within ±1 day of peaks were noted, except for events that were related to linear accelerator sub‐systems such as the treatment couch or the kV cone‐beam CT system, which were ignored. A number of factors, including image ghosting artifacts and actual individual leaf motion were investigated in order to determine the cause of any abnormal behavior.

## 3 RESULTS

### 3.1 Leaf position error

**Figure 1:**
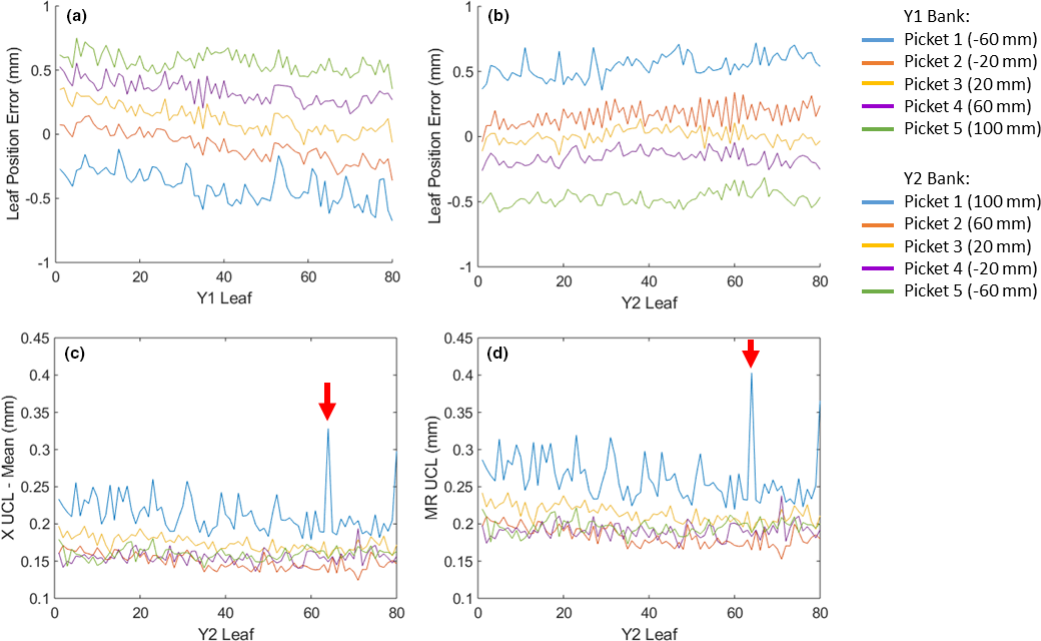
Leaf position error (a) and (b) and upper control limits (UCL), (c) and (d) for each of the 80 multi‐leaf collimator leaves on Y1 and Y2 leaf banks, and each picket, for one unit. Leaf position error was calculated as the mean leaf position over the date range minus the nominal position for that picket (nominal positions for each picket listed in brackets in legend). A negative error indicates that the leaf extended further past the nominal position. (c) Displays Y2 leaf bank individual (X) upper control limits, where mean leaf position was subtracted for ease of comparing between pickets. (d) Displays moving range UCL. The red arrows point to leaf 64, which was exceptionally noisy and thus had larger control limits

Measured leaf position errors for the 13 units included in this work ranged from −1.30 to 1.16 mm over the observation period (mean of −0.01 mm). MLC recalibration due to leaf position error exceeding tolerance (1 mm) was performed once on five units and was required three times on one unit. The minimum time between recalibration for the unit with three MLC recalibrations was 1.5 months. Example average leaf position errors (measured – nominal) per leaf, for one unit, are shown in Figs. 1(a) and 1(b). In general, errors increased with the distance to the radiation isocenter. This can be seen in Fig. 1, where pickets 1 and 5 (the furthest from isocenter) featured the largest offsets. For most of the leaf position errors, opposite leaves appeared to move in the same direction (e.g., a negative shift in Y1 and corresponding positive shift in Y2). As demonstrated in Figs. 1(a) and 1(b), Y1 leaf position errors trended from the most negative offset (picket 1) to the most positive offset (picket 5), while Y2 leaves featured the opposite trend. This was observed in the leaf position errors for all but two units. The increase in measured leaf position error with the distance to the radiation isocenter and the leaf bank motion in the same direction may be explained by a problem with the leaf gain calibration parameter and/or a difference in image pixel scaling factor between the daily MLC QC tests and the image‐based procedure used by the manufacturer for the MLC calibration. Both the daily MLC QC test^6^ and the manufacturer MLC calibration method used an object of known dimensions to determine the image pixel scaling factor. However, it is not possible to compare these two factors, as the manufacturer method does not easily output this result.

**Figure 2:**
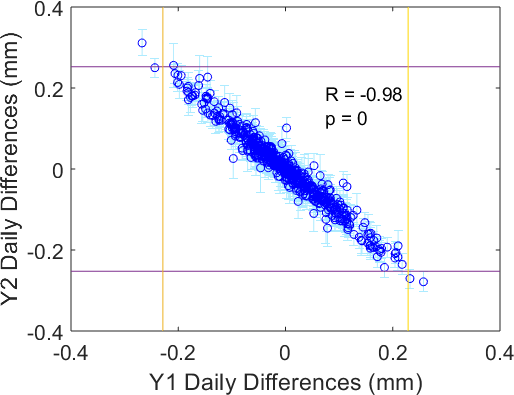
Day‐to‐day differences in measured multi‐leaf collimator position for the Y2 bank vs the Y1 bank, for picket 5 of one unit, averaged over all 80 leaves in each bank. Error bars indicate the standard deviation over all 80 Y2 leaves for that measurement point. Y1 and Y2 moving range upper control limits, averaged over all leaves, are displayed for reference as yellow vertical and purple horizontal lines, respectively. The very strong, significant negative correlation (R = −0.98), along with tight error bars, indicate that Y1 and Y2 leaves appear to shift in the same direction on a day‐to‐day basis, and this trend is common to most leaves. Some of these apparent shifts were greater in magnitude than the moving range control limits, and thus were registered as out‐of‐control points

Strong negative correlations between Y1 and Y2 day‐to‐day differences in leaf position for each picket were observed. Mean correlation coefficients for pickets 1 through 5, averaged over all units (range in brackets), were −0.85 (−0.95 to −0.57), −0.88 (−0.97 to −0.69), −0.78 (−0.97 to −0.06), −0.91 (−0.98 to −0.73), and −0.90 (−0.98 to −0.72). All correlations were significant, except for picket 3 values for 1 of 2 calibration ranges on one unit, and 2 of 4 calibration ranges on another unit (all of which had very few data points). An example correlation plot for picket 5 of one unit is shown in Fig. 2. The non‐zero mean daily differences, in addition to the small standard deviations over all leaves in each bank (as indicated by error bars), demonstrate that these daily shifts are similar for all leaves in a bank. In addition, the magnitudes of some of these daily shifts were greater than the moving range control limits, and thus would register as out‐of‐control behavior.

### 3.2 Control charts

**Figure 3:**
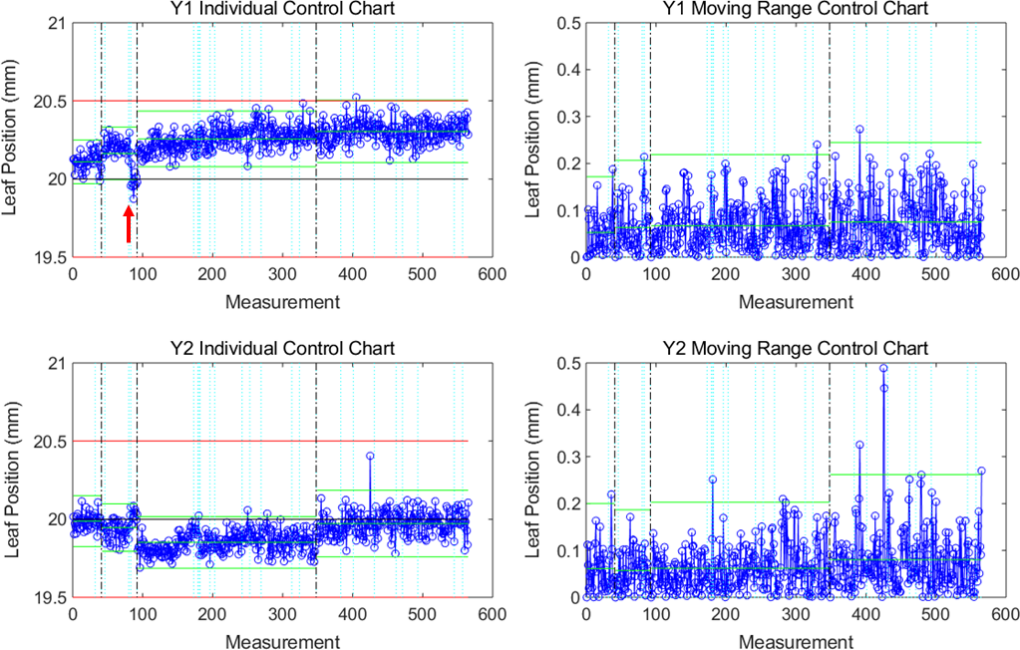
Individual (left) and moving range (right) control charts for a central Y1 leaf and opposing Y2 leaf of one unit, for nominal position = 20 mm (black line). Each measurement (x axis) corresponds to each day the QC test was performed and leaf positions were determined. Green lines indicate control limits, red lines indicate 0.5 mm specification limits, black dotted lines indicate recalibrations, and light blue dotted lines indicate recorded servicing events. The red arrow demonstrates a shift in control at measurement 83 following the replacement of a monitoring ion chamber.

**Figure 4:**
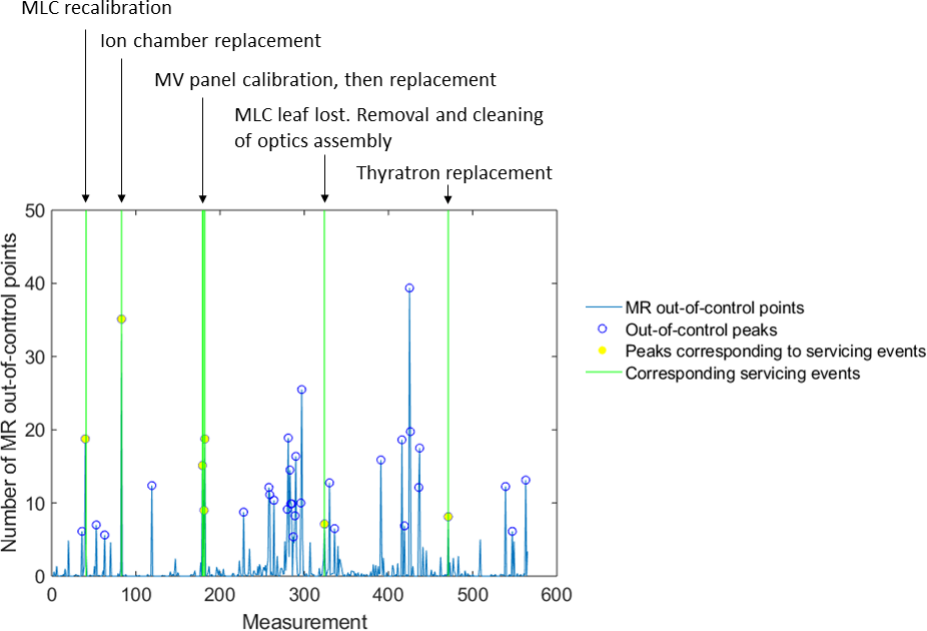
Number of moving range out‐of‐control points, summed over all 160 leaves and all five pickets, plotted over time. Peaks in out‐of‐control (blue circle) were identified as having an amplitude greater than the mean +1 standard deviation in the number of out‐of‐control leaf positions. Yellow circles indicate peaks that corresponded to servicing events displayed above the plot and indicated by green lines (i.e., servicing events that occurred either the measurement day before, same day or day after the peak).

Leaf position varied little over time, as confirmed by very tight individual control limits (mean ±0.19 mm, range 0.09–0.43 mm) and moving range control limits (mean 0.23 mm, range 0.10–0.53 mm). However, the mean in‐control duration was only 2.8 days (range 1–28.5) due to sporadic out‐of‐control events. Figures 1(c) and 1(d) display both individual and moving range control limits for all Y2 MLC leaves for one unit. Variation between leaves was observed; one leaf in particular (leaf 64) had substantially larger control limits due to noisier leaf positioning over time. Manual leaf position detection was performed for leaf 64 on images for a few consecutive days and confirmed that the leaf motion and the test results were consistent. Four other units also featured one or two especially noisy leaves. An example of individual and moving range control charts for a well‐behaved leaf is shown in Fig. 3 and featured distinct shifts in leaf position following recalibration, as well as occasional out‐of‐control points, which were often related to servicing events (Table 1). For example, the shift at measurement 83 in Fig. 3 occurred following replacement of the monitoring ion chamber. Servicing events and machine faults that corresponded to peaks in the number of moving range out‐of‐control leaf positions are listed in Table 1. Figure 4 shows a plot of the number of moving range out‐of‐control leaf positions, peaks in out‐of‐control that were identified using the 1 SD threshold, as well as peaks that corresponded to servicing events for the same unit shown in Fig. 3. For the 13 units included in this work, the most common events that corresponded to out‐of‐control peaks included MV imaging panel calibration (5 occurrences), removal and cleaning of the MLC optics assembly and adjustment of light intensity following a lost leaf (6 occurrences), beam steering and energy adjustment (10 occurrences), as well as preventive maintenance (6 occurrences).

**Table 1:** Servicing events or machine faults that may be related to a shift in control of MLC units

Affected sub‐system	Event description	Frequency
MLC	Lost leaf — optics assembly cleaning and light intensity adjustment	6
	Leaf guide break replacement	3
	Stuck leaf — Lubrication	3
	Leaf guide control board replacement	2
	Mirror replacement	1
Beam steering and energy adjustments	Beam energy adjustment	6
	Beam steering	4
	Beam output adjustment	2
	Magnetron replacement	2
	Monitor ion chamber replacement	1
	Thyratron replacement	1
	Electron gun replacement	1
MV imaging panel	Manufacturer‐recommended panel calibration (dark field and flood field acquisition)	5
	Panel motion mechanism	2
	Panel replacement	1
Collimator	Jaw brake replacement	1
	Collimator rotation detection switch replacement	1
Miscellaneous	Water cooling system and SF6 gas	7
	Preventive maintenance	6

MLC, multi‐leaf collimator; MV, megavoltage.

Servicing events that occurred the day before, day of, or day after a large peak in out‐of‐control points are listed.

**Figure 5:**
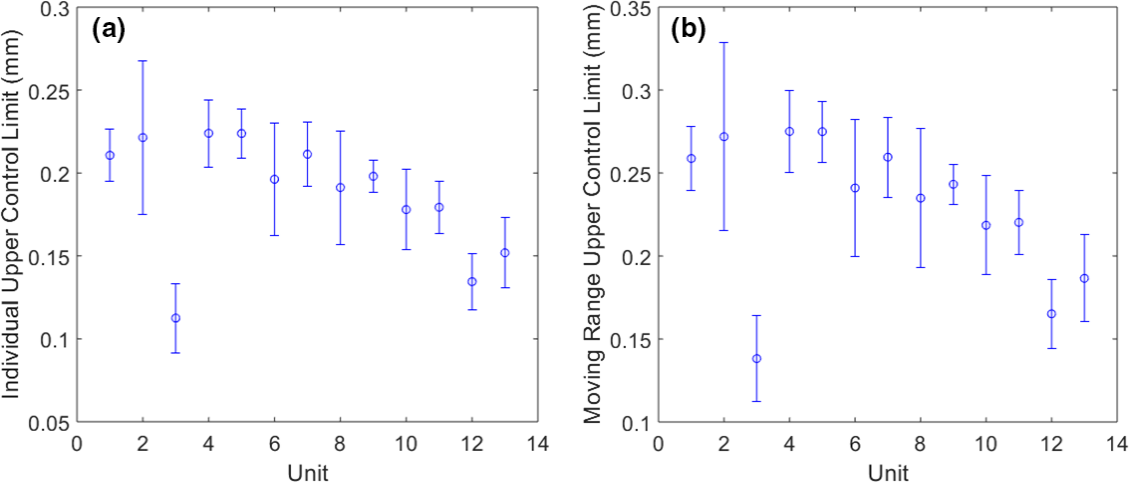
Mean individual (a) and moving range (b) upper control limits for each unit, where error bars indicate standard deviations. Units with the two smallest control limits (unit 3 and 12) correspond to the newest machines

MLC leaf position stability was found to vary between units, as shown by the difference in control limits (Fig. 5). The two units with the tightest control limits were the newest linacs. No correlation was observed between the magnitude of the control limits and the duration of the observation period on a given unit.

### 3.3 Performance within specification levels

**Figure 6:**
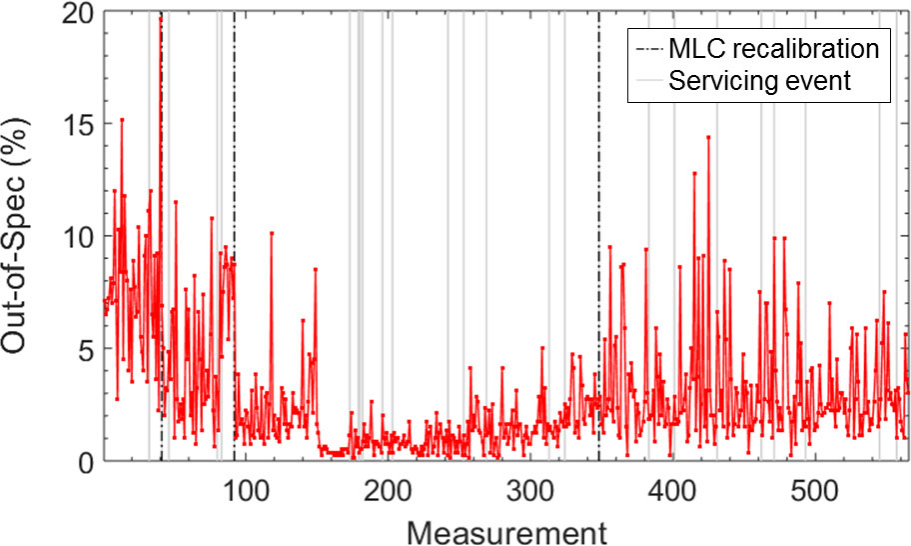
Number of out‐of‐specification (0.5 mm) points relative to total number of points for a single measurement day (n = 800). Out‐of‐specification plots for 1 mm and 1.5 mm levels are not shown, as the out‐of‐specification percentage was essentially zero for all measurements. Dashed lines depict recalibrations, and solid grey lines indicate servicing events.

**Figure 7:**
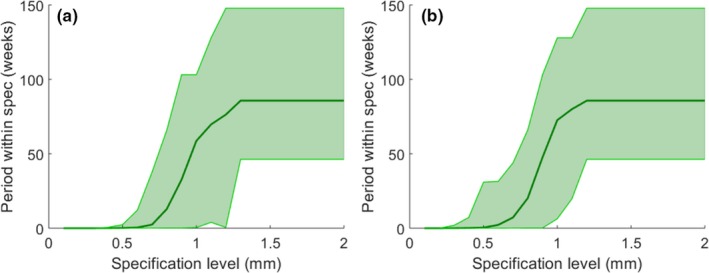
Duration between out‐of‐specification events for each specification level, where the solid line indicates the mean over all units, and light green color wash indicates the range encompassed by the minimum and maximum durations. (a) Includes all leaves. Results improved when leaves 1 and 80 were omitted (b). The average, minimum and maximum in‐specification duration curves plateaued beyond 1.3 mm (a) and 1.2 mm (b) specification level as no out‐of‐specification events were observed for larger specification levels.

Figure 6 demonstrates the daily variations in the relative number of leaf positions that are out‐of‐specification (tolerance ±0.5 mm) for one unit. A plot of duration between out‐of‐specification events vs specification level is shown in Fig. 7. This plot was produced by determining the elapsed time that all measured MLC positions were within specification (or in other words, the duration between subsequent groupings of out‐of‐specification events), for each specification level. Mean, minimum and maximum in‐specification durations were determined for each specification and each unit. The plot summarizes the results of all units, and demonstrates the average, minimum and maximum amount of time a unit’s MLCs could be expected to remain within specification limits, based on our data.

As seen in Fig. 7, measured leaf positions stayed within ±1 mm specification for 457 days on average (range 3–838) and within ±1.5 mm for the entire date range. The average, minimum and maximum in‐specification duration curves plateaued beyond 1.3 mm specification level as no out‐of‐specification events were observed for larger specification levels. For the ±1 mm specification level, there was one outlying duration point (3 days), and the next shortest duration was 28 days, which was bound on one side by the end of data collection. For the majority of units (7 out of 13), there were no out‐of‐specification events for the 1 mm level. The remaining units went out of 1 mm specification one to three times over the entire data collection range. Measurements stayed within ±0.5 mm for 1 day on average (range 0–17); however, our internal requirements for MLC leaf calibration accuracy and to trigger MLC recalibration was 1 mm.

### 3.4 Additional factors influencing leaf position accuracy

The accuracy of leaf edge detection was reduced for the outer leaves 1 and 80, which resulted in slightly greater leaf position errors and control limits. Mean leaf position error was −0.06 and −0.18 mm for leaves 1 and 80, respectively, while all other leaves’ averages fell between ±0.04 mm. Mean individual control limits for leaves 1 and 80 were 0.20 and 0.22 mm, while all other leaves’ means were under 0.19 mm. Mean moving range upper control limits for leaves 1 and 80 were 0.25 and 0.26 mm, while all other leaves’ averages were under 0.24 mm. The most significant impact of the reduced measurement accuracy for leaves 1 and 80 was observed on the calculation of the in‐specification duration. When leaves 1 and 80 were omitted, in‐specification durations improved [Fig. 7(b)]. MLC positions for leaves 2 to 79 never went out of 1 mm specification for the entire period of data collection for 10 of the 13 units. For the remaining three units, leaves 2 to 79 went out of specification only once. However, it is important to note that six units’ MLCs were recalibrated at some point during the data collection range. Although the in‐specification durations increase when excluding leaf 1 and 80, in‐control durations remained the same when omitting these leaves.

**Figure 8:**
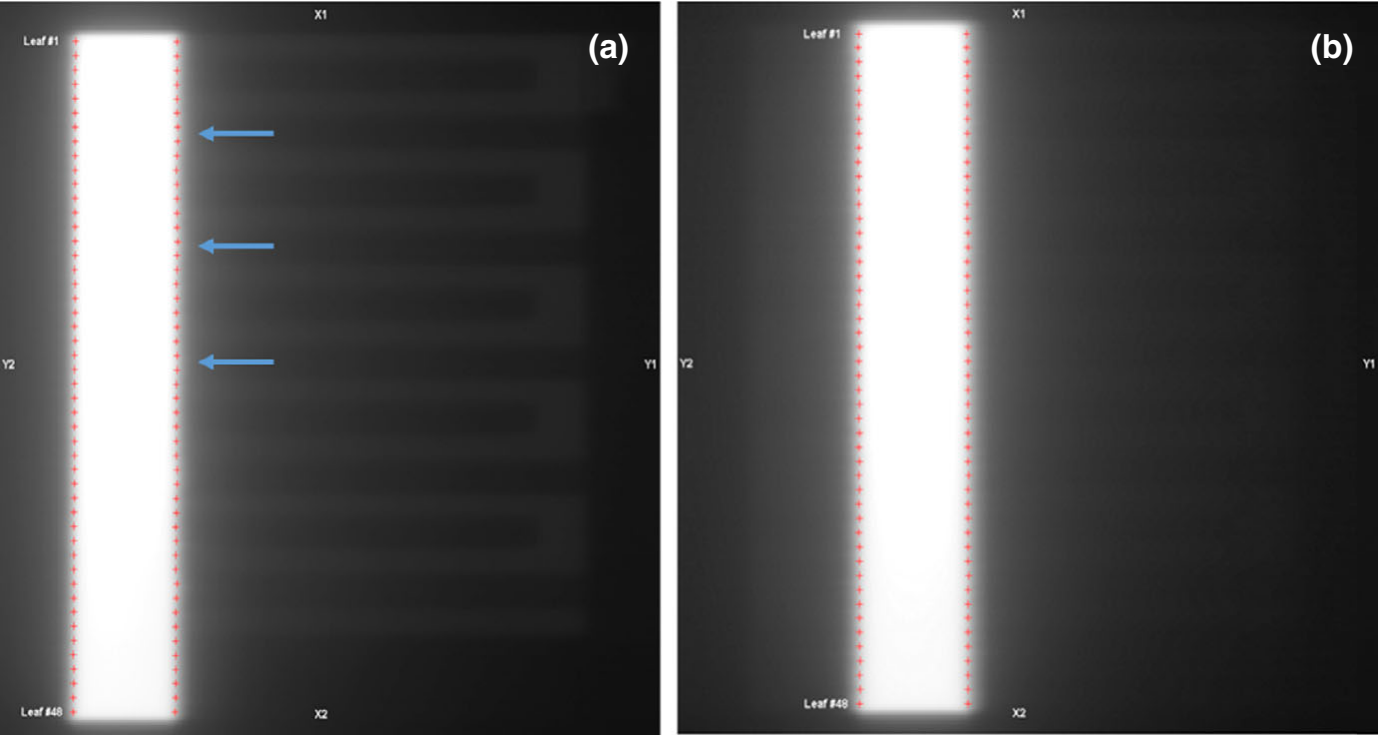
(a) Example picket 1 image, demonstrating ghosting of the prior field’s extended leaf pattern, which impacted leaf position results. Red crosses indicate leaf edge/position, as identified by the automated quality control software. Blue arrows indicate a few example locations where a positive leaf error (i.e., shift right) might be erroneously measured for the Y1 picket due to the presence of the ghosting pattern. The following picket 2 image (b), which does not feature this ghosting pattern, is shown for comparison

In addition to leaves 1 and 80 displaying slightly larger leaf position errors, we also observed noisier measured leaf positions for all units for picket 1. For picket 1, the leaf position errors showed a somewhat periodical pattern [Figs. 1(a) and 1(b)], with sharp peaks occurring every eight or so leaves. Additionally, control limits for picket 1 were often larger than those of other pickets and showed a sinusoidal pattern [Figs. 1(c) and 1(d)]. This additional noise in leaf position for picket 1 was due to an image ghosting artifact from the extended leaf field collected prior to picket 1 leaf measurements (see Fig. 8).

## 4 DISCUSSION

The length of the observation period, the daily test frequency and the number of linacs included in this study enable a thorough evaluation of MLC leaf characteristics and factors that affect their behavior over time. The results of this work have shown that the Elekta Agility MLC performs to a high degree of accuracy and precision over time, which is demonstrated by the tight control limits and duration within a specification level of ±1 mm. However, there were a number of out of control events that were related to certain servicing events. Table 1 identifies servicing events and machine faults that occurred either the day before, day of, or day after large peaks in out‐of‐control leaf positions. Many events were related to the MLCs and MV imaging panel. Because image artifacts such as block artifacts and noisy pixels could impact leaf edge detection, panel recalibration often led to slight variations in measured leaf positions. However, more unexpectedly, other peaks in out‐of‐control events were related to beam adjustments, replacement of key parts such as the electron gun, monitoring ion chamber, thyratron and magnetron, adjustments to other head components, and general maintenance. We do not currently have an explanation for why servicing non‐MLC head components as well as general maintenance were correlated with out‐of‐control MLC behavior. However, we suspect that MLC components might have been jostled during head servicing or that direct MLC servicing or beam steering adjustments performed during general maintenance were not recorded in the service logs. These results indicate that it would be beneficial to perform MLC QC after these servicing events to capture changes in MLC leaf position performance. Depending on the QC results, MLC recalibration could be performed right away after completing the servicing event or MLC performance could be monitored over a few days before deciding if a calibration is required.

A number of factors were believed to contribute to MLC leaf positioning errors measured by this test. For instance, the fact that errors increased with distance to the radiation isocenter may indicate a problem with the leaf gain calibration parameter. It could also result from a difference in image pixel scaling factor between the daily MLC QC tests and the image‐based procedure used by the manufacturer for MLC calibration. The strong, significant negative correlations between Y1 and Y2 mean daily differences in leaf position could be explained by beam spot motion, since Y1 and Y2 leaves appear to move in the same direction on average. In addition, the small standard deviations over leaves in a leaf bank demonstrate that these trends were common to most, if not all leaves. This is consistent with the beam spot motion theory, since any motion would cause the projection of all Y1 and Y2 leaves on the portal imager to appear to move in the same direction from 1 day to the next. For most pickets on most units, the magnitudes of some mean daily differences were even greater than the moving range control limits. In fact, when mean daily differences were plotted over time for each picket, peaks in mean daily differences were found to correspond to peaks in moving range out‐of‐control events. Thus, beam spot motion is likely the cause of some of the observed out‐of‐control behavior of MLC leaves. Monitoring beam steering parameters while running the MLC test could potentially help detect beam spot motion and would confirm its impact on MLC leaf positions.

Since leaf positions are extracted from portal images, they can be impacted by image artifact. Picket 1 images featured a ghosting pattern from the prior extended leaf field (Fig. 8), which resulted in noisier leaf positions over time, larger control limits, and leaf position errors that demonstrated a periodical trend across leaves (Fig. 1). We have been investigating methods to reduce the ghosting, including changing the order in which images are acquired, or allowing a time delay in between the extended leaf and picket 1 fields. When employing these strategies, the ghosting was reduced and picket 1 leaf patterns were more similar to other pickets.

This test also revealed variation between MLC leaves within a single bank, and in particular a few leaves were found to be exceptionally noisy. After further investigation, it was determined that the noisy leaf measurements were in fact real, and these few leaves were moving more than others. While the positioning accuracy and stability for some noisy leaves improved after MLC recalibration, other leaves then became noisy. The MLC test was clearly able to detect unstable leaves, but the ability to improve positioning performance for all leaves seems limited to the replacement of the linac leaf control electronics.

Finally, we believe the test was less accurate at detecting the position of leaves 1 and 80. This was demonstrated by greater leaf position errors and larger control limits. In‐specification durations were improved by omitting these leaves from analysis (see Fig. 7). The combination of penumbra on two adjacent edges of the leaf could have reduced the accuracy of leaf edge detection at the leaf tip. It is possible that bringing the jaw in to cover the outer edge of these leaves could reduce the second edge penumbra and improve accuracy of detection.

The current recommended tolerance for monthly MLC leaf positioning accuracy is ±1 mm.^1^ Our data indicates that majority of units never fall outside of this tolerance. For this reason, we believe that a tolerance of ±1 mm is sufficient, if not overly generous, for most Agility MLC units. It is important to note, however, that the in‐specification durations reported in Fig. 6(a) are somewhat influenced by the MLC leaf position tolerance of ±1 mm employed in our center, as recalibration is only performed when errors surpass this threshold. To more accurately evaluate in‐specification durations for each specification level, one would have to adjust recalibration accuracy and frequency accordingly. With proper MLC calibration and correction of image artifacts, along with higher frequency MLC testing, we believe that the Elekta MLC model may be capable of performing to a specification level tighter than ±1 mm, although this needs to be confirmed with further testing. If a ±1 mm tolerance is employed, our data indicates that a monthly testing frequency is a conservative choice but is appropriate as multiple type of servicing events can impact leaf position accuracy. The methodology and the in‐specification duration data (Fig. 7) presented in this work provide the basis for determining an appropriate QC test frequency, if a certain level of MLC performance is desired. While 1 mm leaf positioning accuracy may be sufficient for current target setup margins, it is possible that sub‐millimeter MLC performance may be beneficial for hypo‐fractionated SBRT treatments that use tight margins (e.g., in the range of 1–3 mm). The current automated QC test is performed only at 0° gantry angle. The motivation for this was to demonstrate the test’s value in its most accurate form, removing imaging panel and gantry sag as potential confounders to measured leaf positions. However, TG 142 recommends testing MLC leaf positioning at all four cardinal gantry angles.^1^ The accuracy of leaf position measurement at other gantry angles will be explored in future work.

Although initially utilized mainly in the manufacturing industry, the use of statistical process control tools for monitoring and characterizing process performance is slowly becoming more common in the field of radiation therapy. Studies have used these tools to evaluate patient‐specific IMRT QA and monitor unit verification,^8^, ^9^, ^10^, ^11^ output and beam flatness/symmetry measurements,^12^ electron spectra from linear accelerators,^13^ and MLC QA.^6^ Some groups even advocate to replace traditional specification‐based QA with control‐chart driven quality management.^14^ While we have demonstrated a retrospective use of statistical process control tools to analyze this large amount of data and we have been able to relate some servicing events with MLC out‐of‐control behavior, there are still a lot of observed out‐of‐control events that remained unexplained. Linac servicing events in our institutions are recorded manually in a service database. We believe that the use of control charts to detect change in MLC leaf position performance should be coupled to a more exhaustive method to record servicing events in order to facilitate the investigation of out‐of‐control behavior. In addition, automated recording of machine operating parameters such as beam steering parameters and MLC optical chain parameters could help understanding changes in MLC performance observed during QC. The combination of recording servicing and machine operation parameters, along with the daily MLC QC test analyzed with control charts, could lead to optimized MLC leaf position performance and inform the user on when MLC recalibration is required.

## 5 CONCLUSION

The Elekta Agility MLC model was found to perform with high stability, as evidenced by the tight control limits. The in‐specification durations support the current recommendation of monthly MLC QC tests with a ±1 mm tolerance in order to detect potential drifts in performance and apply appropriate corrective action. Multiple factors were found to influence leaf positioning accuracy, including beam spot motion, leaf gain calibration, drifting leaves, and image artifacts. In particular, out‐of‐control leaf positions were often correlated to servicing events, indicating that certain types of servicing events may require subsequent MLC calibration. Future work is on‐going to determine if Agility performance can be optimized further using high‐frequency QC test results to drive recalibration frequency.

## CONFLICT OF INTEREST

No conflict of interest.
